# Estimation of postpartum depression risk from electronic health records using machine learning

**DOI:** 10.1186/s12884-021-04087-8

**Published:** 2021-09-17

**Authors:** Guy Amit, Irena Girshovitz, Karni Marcus, Yiye Zhang, Jyotishman Pathak, Vered Bar, Pinchas Akiva

**Affiliations:** 1 KI Research Institute, Kfar Malal, Israel; 2 Division of Health Informatics, Department of Population Health Sciences, Weill Cornell Medicine, New York, NY USA; 3 Women’s Mental Health, Sheba Medical Center, Ramat Gan, Israel

**Keywords:** Postpartum depression, Machine learning, Electronic health records

## Abstract

**Background:**

Postpartum depression is a widespread disorder, adversely affecting the well-being of mothers and their newborns. We aim to utilize machine learning for predicting risk of postpartum depression (PPD) using primary care electronic health records (EHR) data, and to evaluate the potential value of EHR-based prediction in improving the accuracy of PPD screening and in early identification of women at risk.

**Methods:**

We analyzed EHR data of 266,544 women from the UK who gave first live birth between 2000 and 2017. We extracted a multitude of socio-demographic and medical variables and constructed a machine learning model that predicts the risk of PPD during the year following childbirth. We evaluated the model’s performance using multiple validation methodologies and measured its accuracy as a stand-alone tool and as an adjunct to the standard questionnaire-based screening by Edinburgh postnatal depression scale (EPDS).

**Results:**

The prevalence of PPD in the analyzed cohort was 13.4%. Combing EHR-based prediction with EPDS score increased the area under the receiver operator characteristics curve (AUC) from 0.805 to 0.844 and the sensitivity from 0.72 to 0.76, at specificity of 0.80. The AUC of the EHR-based prediction model alone varied from 0.72 to 0.74 and decreased by only 0.01–0.02 when applied as early as before the beginning of pregnancy.

**Conclusions:**

PPD risk prediction using EHR data may provide a complementary quantitative and objective tool for PPD screening, allowing earlier (pre-pregnancy) and more accurate identification of women at risk, timely interventions and potentially improved outcomes for the mother and child.

**Supplementary Information:**

The online version contains supplementary material available at 10.1186/s12884-021-04087-8.

## Background

Postpartum depression (PPD) is one of the most common complications of childbearing, estimated to affect 10–15% of mothers worldwide, with higher incidence rates in developing countries [1]. PPD is a leading cause of maternal perinatal mortality, accounting for ~ 20% of postpartum deaths [2]. There are also negative associations between PPD symptoms and mother-baby bonding, infant physical and cognitive development, language development, infant behaviors and quality of sleep [3].

The strongest risk factor for PPD is prior history of mood and anxiety problems and, in particular, untreated depression and anxiety during pregnancy [4]. Additional risk factors include stressful life events, pregnancy and childbirth complications, lack of emotional support from spouse or family and problems of alcohol/drug abuse [5].

Routine screening for PPD is broadly based on identifying symptoms using self-reported questionnaires such as the Edinburgh Postnatal Depression Scale (EPDS) [6] or Patient Health Questionnaire 9 (PHQ-9) [7]. These are often administered at the 6–8 weeks postpartum examination, which may be well after the onset of the condition [8]. Another limitation of self-reported questionnaires, which may affect their predictive accuracy, is that responses depend on self-disclosure and overcoming personal and social stigma [9]. The reported accuracy of EPDS in predicting PPD varies considerably. In a systematic review [10], the pooled sensitivity and specificity of 8 studies were 0.80 and 0.81, respectively.

It was reported that up to 50% of women who develop PPD began experiencing symptoms during pregnancy, and in some cases, as early as in first trimester [11]. However, risk of PPD is often overlooked during the pregnancy follow-up process, which is focused on the physical health of the mother and the well-being of the fetus. It is well-accepted that intervention by either pharmacological treatment or psychological counseling may reduce the risk of perinatal depression [12] and may improve the outcomes of both mother and child [13], however the safety of exposure to antidepressant medications during pregnancy and breastfeeding is an area of current research [14] . Although the risk factors of PPD are well-known, there are no quantitative risk assessment tools to support the screening and clinical management of women during perinatal period. Electronic health records (EHR) from primary care services provide rich representation of the patient’s medical condition, including diagnoses, drug prescriptions, procedures and lab tests. This information, along with the patient’s socio-demographic background are a useful source of information for estimating risk and predicting disease. Machine learning is a powerful computational data analysis tool, suitable for deriving insights from large sets of multivariate medical data such as EHR. The motivation for this work is to develop an automated tool for PPD risk surveillance using data from EHR. Such tool should utilize machine learning to effectively combine a multitude of parameters providing an objective assessment of PPD risk that may enable early interventions and improved outcomes. Previous work on data-driven risk prediction of PPD used relatively small cohorts. One example is an artificial neural network, trained to predict PPD in a group of 1397 women (160 positive), reported to achieve sensitivity and specificity of 0.84 and 0.81, respectively [15]. Various machine learning classifiers were reported to achieve area under the receiver operator characteristic curve (AUC) ranging from 0.79 to 0.89 on a larger validation cohort of 53,972 patients at multiple sites [16]. Both these previous studies analyzed EHR from a hospital network. In such clinical settings patients that receive their obstetrics and gynecology care at the hospital may use a different provider for their mental health services. This limits the availability of reliable outcome measures and may introduce biases. Recently, a PPD prediction model using primary care EHR was reported to achieve AUC of 0.71 on a nationwide cohort [17]. The rate of PPD in this cohort was 1.9%, which is lower than the estimated population level prevalence.

In the current work we aim to study the potential use of primary care data from EHR in predicting PPD and identifying patients at risk during the 12 month period after childbirth, by applying machine learning techniques on a very large cohort of over 260K subjects. We demonstrate the value of EHR-based prediction in early identification of women at risk, and in augmenting the predictive accuracy of the standard EPDS screening tool. To the best of our knowledge, this study is the first to suggest combining an EHR-based risk model with EPDS score in order to improve PPD screening.

## Methods

We analyzed primary care EHR data from IQVIA Medical Research Data (IMRD), incorporating data from The Health Improvement Network (THIN, a Cegedim database). The dataset contains records of over 18 million patients (over 3 million active patients), covers approximately 5% of the UK population, and is representative of the population in terms of demographics and major condition prevalence [18]. The data includes patient demographics, medical diagnoses, drug prescriptions, anthropometric measurements and lab tests. Pregnancies resulting in a live birth were identified by searching for recorded medical codes, following the logic described by Matcho et al. [19].

Our cohort included women between the ages of 18 and 45 who had their first live birth between 2000 and 2017. Non-first deliveries were not included in order to avoid bias by multiple inclusions of patients, so that the cohort is more homogeneous and PPD is not used as both covariate and outcome. We included subjects whose medical file was “active” (at least one recorded diagnosis, one drug prescription and one lab test) during the period of the pregnancy, as well as during the preceding 2 years and the year following delivery.

PPD outcome was defined based on the occurrence of one of the following in the EHR data during the 12 month period after childbirth [20]: (1) diagnosis of depression; (2) new treatment with antidepressant drug; and (3) non-pharmacological treatment for depression. Patients who had records of antidepressant prescriptions both before (within 1 year before the pregnancy) and after giving birth, and did not have explicit diagnosis of depression during the postpartum period, were excluded from the analysis due to the ambiguity in determining their outcome.

The typical methodology of utilizing machine learning algorithms for risk prediction tasks is to use a subset of the data for optimizing a statistical model, while reserving the remaining data for evaluating the model’s performance [21]. These subsets, referred to as training and testing sets, respectively, can be selected either randomly or by some criteria that emulates a real-life scenario. For example, using earlier and later periods of time, or using different geographical regions for the training and testing subsets.

**Fig. 1:**
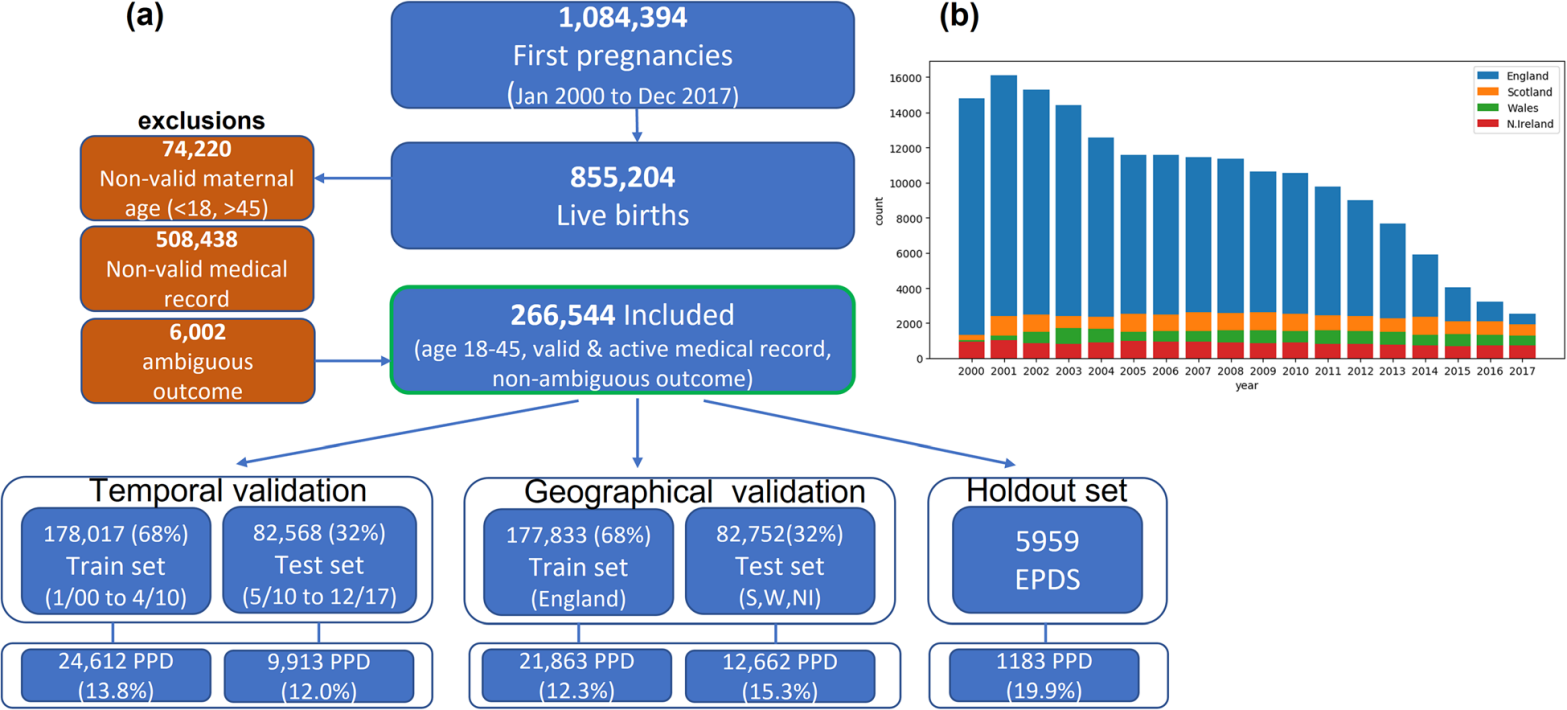
Cohort generation flow, with partitioning into training and testing sets using either geographical or temporal criteria (**a**), and the distribution of the cohort by year and by country (**b**)

Patient data were split into training and testing sets (Fig. 1) containing approximately 2/3 and 1/3 of the patients, respectively. We used three different validation schemes: (1) Geographical validation, using England patients for training and Scotland, Wales and Northern Ireland patients for testing; (2) Temporal validation, using earlier deliveries from January 2000 until April 2010 for training, and later deliveries for testing; and (3) Random split (pooled 3-fold cross validation).

We defined an additional ‘holdout’ test set, of women who had EPDS scores recorded in their EHR data. This information was not available for all patients because EPDS in the UK is administered at the discretion of the primary care physician, rather than used as a screening tool. This subset was used to evaluate the potential additive value of EHR-based risk prediction to the EPDS score.

For each patient we extracted a multitude of variables from the following categories: (1) Demographic, socio-economic and personal measures (age, ethnicity, marital status, deprivation index, pre-pregnancy BMI, habits of smoking, alcohol use and drug use); (2) Medical diagnoses during pregnancy (mental disorders and symptoms, pregnancy complications, other relevant health conditions; (3) Labor complications (cesarean section, episiotomy) and infant-related measures (gestational week, birth weight, APGAR score); (4) History of medical diagnoses within 2 years before the pregnancy; (5) Drug prescriptions during and prior-to pregnancy (antidepressants, antibacterials, antihistamines, beta-blocking agents); (6) Healthcare utilization, measured by counts of visits, diagnoses and drug prescriptions during and prior-to pregnancy. Early prediction of PPD was assessed by training the classifiers using only pre-pregnancy variables. Missing values of continuous variables were replaced using mean imputation. Categorical variables were converted to binary variables by replacing each variable *x* that can take values {*v*_1_,…,*v*_n_} with *n* binary variables *x*_1_,*x*_2_,…,*x*_n_, such that *x*_i_ = 1 if *x* = *v*_i_, and *x*_i_ = 0 otherwise (one-hot encoding). The clinical codes used to define diagnoses and drug variables are given in the supplementary appendix.

All models were trained using gradient tree boosting algorithm [22], a supervised learning technique based on iteratively optimizing the predictive value of an ensemble of decision trees. The prediction performance was measured by the area under the receiver operator characteristics curve (AUC), as well as by the sensitivity achieved at fixed specificity of 0.80. McNemar’s test was used to compare the performance of different predictors. Variable importance was analyzed using Shapley additive explanations (SHAP), a game theoretic approach to explain the output of machine learning models [23].

## Results

Overall, 266,544 women met the inclusion criteria (Fig. 1). A subset of 5959 women, who had EPDS scores recorded in their EHR data, were used as a holdout test set, to evaluate the potential additive value of EHR-based risk prediction to the EPDS score.

The PPD outcome criteria was met by 35,708 patients (13.4%). As shown in Table 1, 45.6% of the PPD-positive patients had records of both depression diagnosis and treatment, 38% had recorded treatment without diagnosis and 16.4% had recorded diagnosis without treatment. The PPD prevalence in each of the training and testing sets ranged from 12 to 20%.

**Table 1:** Prevalence of PPD outcome in the study cohort, by diagnosis of depression (Dx) and by treatment for depression (Tx)

	Recorded Depression Tx	No recorded Depression Tx	Total(% patients)
Recorded Depression Dx	**16,284** (45.6% of PPD)	**5869** (16.4% of PPD)	22,153 PPD by Dx (8.3% of cohort)
No recorded Depression Dx	**13,555** (38.0% of PPD)	**230,836** non-PPD (86.6% of cohort)	
Total (% pts)	29,839 PPD by Tx (11.2% of cohort)		35,708 PPD (13.4% of cohort)

The baseline characteristics of the entire cohort, as well as the train, test and holdout subsets are shown in Table 2. The average maternal age at childbirth was 30.0 ± 5.8 years and the pre-pregnancy BMI was 25.0 ± 5.4. Previous history of depression was recorded in 6.5% of the cohort, with higher prevalence of 8.6% in the holdout set of subjects with recorded EPDS scores, which may indicate that this subset is biased towards patients at higher risk for PPD. The mean time from the delivery to the event determining the PPD outcome (diagnosis or treatment) was 150 ± 100 days. In the holdout set, the mean time for administrating the first EPDS questionnaire was 77.5 ± 65.8 days following labor. The temporal and geographical distributions of the data (Fig. 1b) showed a trend of decrease in the number of subjects in the cohort throughout the years, mostly in England, which represents changes in the content of the dataset.

**Table 2:** Cohort’s characteristics

		Temporal validation		Geographical validation		
Characteristic	All (%)	Train set (01/00–04/10)	Test set (05/10–12/17)	Train set (E)	Test set (S,W,NI)	Holdout set
N	266,544	178,017 (68)	82,568 (32)	177,833 (68)	82,752 (32)	5959
Age	30.0 ± 5.8	30.1 ± 5.8	29.8 ± 5.8	30.3 ± 5.7	29.5 ± 5.8	30.2 ± 5.7
**Ethnicity**						
White	99,971 (37.5)	57,295 (32.1)	40,600 (49.2)	71,666 (40.3)	26,229 (31.7)	2076 (34.8)
Asian	7367 (2.8)	4095 (2.3)	3214 (3.9)	6667 (3.7)	642 (0.8)	58 (1.0)
Black	3167 (1.2)	1708 (1.0)	1441 (1.7)	2968 (1.7)	181 (0.2)	18 (0.3)
Other	2412 (0.9)	1110 (0.6)	1276 (1.5)	1837 (1.0)	549 (0.7)	26 (0.4)
Unknown	152,573 (57.2)	113,334 (63.7)	35,467 (43.0)	93,737 (52.7)	55,064 (66.5)	3772 (63.3)
**Marital status**						
Single	34,145 (12.8)	20,612 (11.6)	12,567 (15.2)	16,118 (9.1)	17,061 (20.6)	966 (16.2)
Married	62,929 (23.6)	43,526 (24.5)	17,460 (21.1)	40,765 (22.9)	20,221 (24.4)	1943 (32.6)
Unknown	169,470 (63.6)	113,879 (64.0)	52,541 (63.6)	120,950 (68.0)	45,470 (54.9)	3050 (51.2)
Country						
England	182,506 (68.5)	129,113 (72.5)	48,720 (59.0)	177,833 (100)	0	4673 (78.4)
Scotland	42,113 (15.8)	23,982 (13.5)	16,984 (20.6)	0	40,966 (49.5)	1147 (19.2)
Wales	26,565 (10.0)	15,481 (8.7)	10,963 (13.3)	0	26,444 (32.0)	121 (2.0)
N. Ireland	15,360 (5.8)	9441 (5.3)	5901 (7.1)	0	15,342 (18.5)	18 (0.3)
Deprivation index quantile	3.03 ± 1.3	2.96 ± 1.3	3.22 ± 1.2	2.95 ± 1.3	3.24 ± 1.2	2.7 ± 1.3
Pre-pregnancy BMI	25.0 ± 5.4	24.9 ± 4.2	25.2 ± 4.9	24.9 ± 4.2	25.2 ± 4.6	25.1 ± 4.5
Cesarean section	51,151 (19.2)	30,724 (17.2)	19,195 (23.2)	31,531 (17.7)	18,388 (22.2)	1232 (20.7)
Smoking	64,778 (24.3)	44,807 (25.2)	18,482 (22.4)	41,792 (23.5)	21,497 (26.0)	1489 (25.0)
History of depression	17,384 (6.5)	12,495 (7.0)	4379 (5.3)	12,052 (6.8)	4822 (5.8)	510 (8.6)

**Fig. 2:**
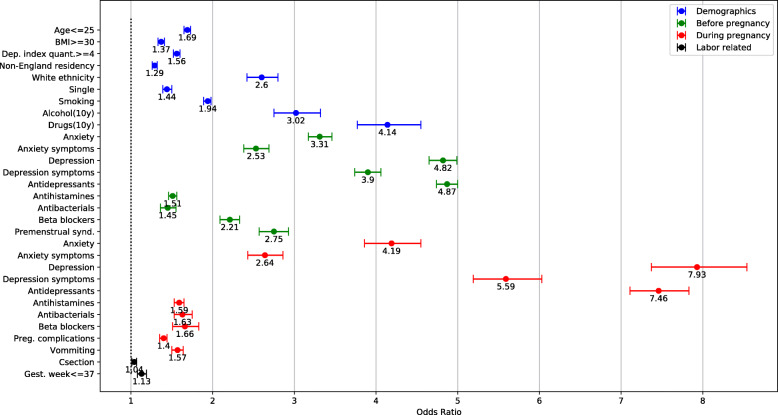
PPD odds ratios of single predictor variables, including socio-demographic, diagnoses, drug prescriptions and labor-related variables. Horizontal error bars indicate 95% confidence intervals

A bivariate analysis comparing the PPD and the non-PPD groups (Table 3, Fig. 2), indicated the significance of mental-health related variables such as previous diagnosis of depression, depression symptoms, or prescribed antidepressants during or prior to the pregnancy, with unadjusted odds ratios (OR) and their 95% confidence intervals (CI) ranging from 3.9 (CI 3.7,4.1) to 7.9 (CI 7.4,8.5). Additional single variables with high odds ratios were drug or alcohol abuse during 10 years prior to the pregnancy (OR = 4.1 (CI 3.8,4.6) and 3.0 (CI 2.8,3.3), respectively), history of premenstrual syndrome (OR = 2.7 (CI 2.6,2.9)) and smoking (OR = 1.9 (CI 1.9,2.0)).

**Table 3:** Predictive variables. For continuous variables, the numbers indicate average and standard deviation, with *P*-value of an independent t-test. For binary variables, the number of occurrences and their percentage (out of the subjects with a ‘True’ value) are given, along with the unadjusted odds ratio (OR) of PPD with 95% confidence intervals (CI)

Variable	PPD (***N*** = 35,708; 13.4%)	Non-PPD (***N*** = 230,836; 86.6%)	OR (CI) /***P***-value
Age (yrs)	28.9 ± 6.1	30.2 ± 5.7	*P* < 0.001
Age ≤ 25 yrs	10,634 (18.7)	46,330 (81.3)	1.69 (1.65,1.73)
Pre-pregnancy BMI	25.7 ± 5.9	24.9 ± 5.3	*P* < 0.001
BMI > =30	5016 (17.3)	24,034 (82.7)	1.44 (1.39,1.48)
Deprivation index	3.28 ± 1.28	3.00 ± 1.27	*P* < 0.001
Deprivation index quantile > =4	15,257 (16.7)	76,360 (83.3)	1.56 (1.52,1.60)
Resides in England (vs. non-England)	22,714 (12.4)	159,792 (87.6)	0.78 (0.76,0.80)
White ethnicity (vs. known non-white)	13,609 (13.6)	86,362 (86.4)	2.60 (2.42,2.80)
Married (vs. known single)	7171 (11.4)	55,758 (88.6)	0.69 (0.67,0.72)
Smoking (currently)	12,847 (19.8)	51,931 (80.2)	1.94 (1.89,1.98)
Alcohol abuse (10y)	634 (31.6)	1374 (68.4)	3.02 (2.75,3.32)
Drug abuse (10y)	714 (38.7)	1131 (61.3)	4.14 (3.77,4.55)
**During pregnancy:**			
Anxiety	950 (38.8)	1497 (61.2)	4.19 (3.86,4.54)
Anxiety symptoms	831 (28.7)	2064 (71.3)	2.64 (2.43,2.86)
Depression	1579 (54.1)	1339 (45.9)	7.93 (7.37,8.54)
Depression symptoms	1281 (45.6)	1526 (54.4)	5.59 (5.19,6.03)
Antidepressants	3508 (51.4)	3322 (48.6)	7.46 (7.11,7.83)
Antihistamines	4257 (19.0)	18,130 (81.0)	1.59 (1.53,1.64)
Antibacterials	1109 (20.0)	4439 (80.0)	1.63 (1.53,1.75)
Beta blockers	557 (20.4)	2178 (79.6)	1.66 (1.51,1.83)
Pregnancy complications	6033 (17.1)	29,351 (82.9)	1.40 (1.35,1.44)
Vomiting	2354 (19.2)	9936 (80.8)	1.57 (1.50,1.64)
**Before pregnancy (2y)**			
Anxiety	3008 (32.5)	6239 (67.5)	3.31 (3.17,3.46)
Anxiety symptoms	1463 (27.6)	3831 (72.4)	2.53 (2.38,2.69)
Depression	5702 (39.4)	8764 (60.6)	4.82 (4.65,4.99)
Depression symptoms	3902 (35.7)	7041 (64.3)	3.90 (3.74,4.06)
Antidepressants	12,024 (35.6)	21,797 (64.4)	4.87 (4.74,5.00)
Antihistamines	5657 (18.1)	25,604 (81.9)	1.51 (1.46,1.56)
Antibacterials	1163 (18.2)	5237 (81.8)	1.45 (1.36,1.55)
Beta blockers	1960 (24.9)	5920 (75.1)	2.21 (2.09,2.33)
Premenstrual syndrome (10y)	1311 (29.3)	3161 (70.7)	2.74 (2.57,2.93)
**Labor-related**			
Cesarean section	7051 (13.8)	44,100 (86.2)	1.04 (1.01,1.07)
Gestational week	39.66 ± 2.25	39.79 ± 2.12	*P* < 0.001
Gest. week<=37	2141 (14.6)	12,569 (85.4)	1.13 (1.08,1.19)
APGAR 1 min	8.32 ± 1.6	8.44 ± 1.6	*P* < 0.001
APGAR 5 min	9.29 ± 1.0	9.36 ± 0.9	*P* < 0.001

**Fig. 3:**
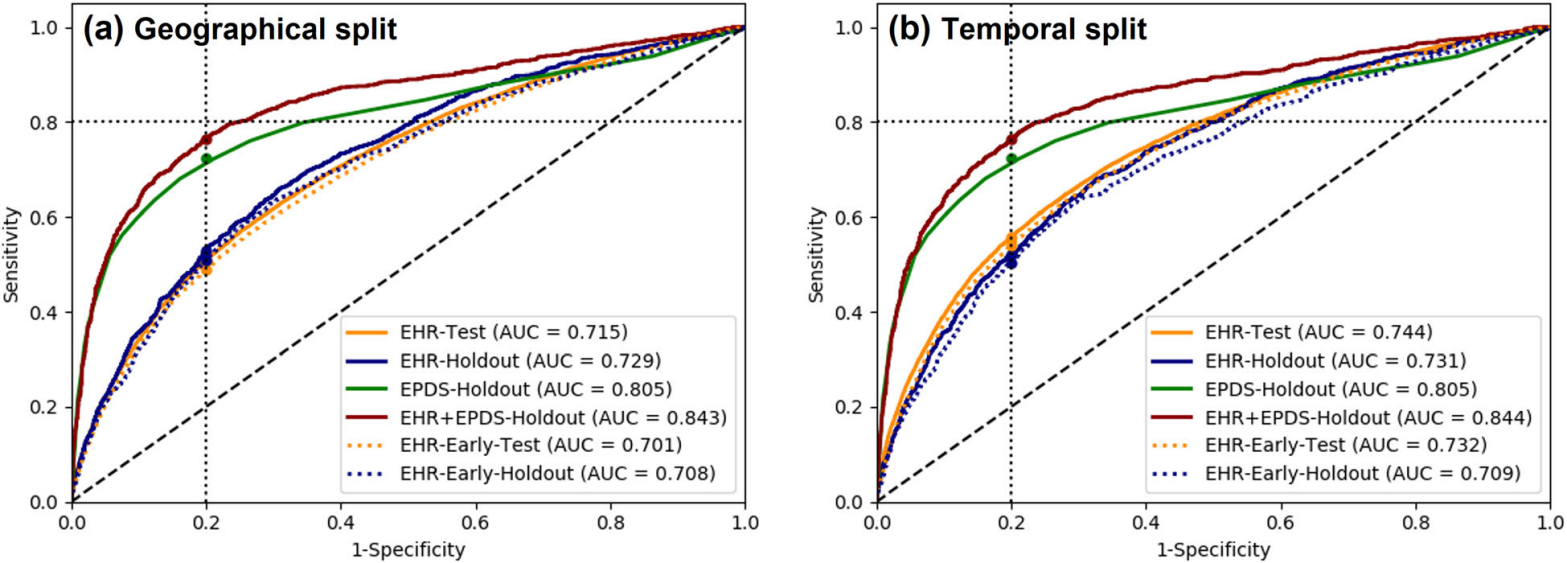
Performance of PPD prediction models with geographical split (**a**) and temporal split (**b**) of the training/testing sets. The models are compared by the area under the ROC curve (AUC) and by the sensitivity at false positive rate of 0.2. EHR: risk score based on Electronic Health Records; EPDS: score based on Edinburgh questionnaire

The full EHR-based prediction model included 69 variables. The prediction performance of this model on different data subsets are shown in Table 4 and the ROC curves are illustrated in Fig. 3. The model’s AUC was 0.715, 0.74 and 0.73 on the geographical-, temporal- and random-split test sets, respectively. On the holdout set, combining the EHR-based prediction score with the EPDS score (by normalized addition) improved the AUC from 0.805 (EPDS alone) to 0.844 (combined, *P* < 0.001) and the sensitivity from 0.72 to 0.76, at specificity of 0.80 (*P* < 0.001). The performance of EHR-based model in early prediction of PPD risk, using only pre-pregnancy variables, was slightly inferior to the full model, with an AUC drop ranging between 0.01 and 0.02. On a subgroup of patients (*N* = 223,681) without recorded history of mental illness (including depression, psychoses, personality disorders or antidepressant prescriptions), the EHR-based prediction achieved AUC of 0.67.

**Table 4:** Prediction results using different prediction models and validation strategies. EHR: risk score based on Electronic Health Records; EPDS: score based on Edinburgh questionnaire; AUC: area under the ROC curve; CI: 95% confidence intervals

Prediction model	Configuration	Test data	N (prevalence)	AUC (CI)	Sensitivity @0.80specificity (CI)
EHR	Geographical(train: England)	Test set (S,W,NI)	82,752 (0.15)	0.715 (0.709,0.719)	0.509 (0.499,0.518)
		Holdout set	5959 (0.20)	0.729 (0.711,0.746)	0.530 (0.495,0.562)
		England	4673 (0.18)	0.729 (0.709,0.748)	0.534 (0.498, 0.571)
		Non-England	1286 (0.26)	0.712 (0.677,0.744)	0.489 (0.416,0.565)
EPDS score		Holdout set	5959 (0.20)	0.805 (0.787,0.821)	0.723 (0.695,0.750)
		England	4673 (0.18)	0.825 (0.807, 0.842)	0.732 (0.671,0.773)
		Non-England	1286 (0.26)	0.771 (0.736,0.805)	0.688 (0.637,0.738)
EHR + EPDS		Holdout set	5959 (0.20)	0.843 (0.828,0.856)	0.764 (0.736,0.791)
		England	4673 (0.18)	0.860 (0.846,0.876)	0.772 (0.740,0.804)
		Non-England	1286 (0.26)	0.815 (0.783,0.844)	0.727 (0.677,0.776)
EHR	Temporal(train: 1/00–4/10)	Test set (05/10–12/17)	82,568 (0.12)	0.744 (0.739,0.749)	0.558 (0.548,0.569)
		Holdout set	5959 (0.20)	0.731 (0.714,0.748)	0.521 (0.489,0.553)
		1/00–4/10	4779 (0.18)	0.738 (0.719,0.757)	0.534 (0.494,0.572)
		5/10–12/17	1296 (0.28)	0.689 (0.655,0.723)	0.475 (0.407,0.538)
EPDS score		Holdout set	5959 (0.20)	0.805 (0.787,0.821)	0.723 (0.695,0.750)
		1/00–4/10	4779 (0.18)	0.775 (0.754,0.795)	0.677 (0.646,0.71)
		5/10–12/17	1296 (0.28)	0.872 (0.847,0.895)	0.821 (0.773,0.866)
EHR + EPDS		Holdout set	5959 (0.20)	0.844 (0.83,0.857)	0.764 (0.735,0.791)
		1/00–4/10	4779 (0.18)	0.823 (0.806,0.841)	0.733 (0.697,0.768)
		5/10–12/17	1296 (0.28)	0.886 (0.866,0.907)	0.836 (0.797,0.876)
EHR	Pooled 3-fold crossvalidation	Random test set	86,862 (0.13)	0.732 (0.729,0.735)	0.535 (0.53,0.541)
EHR-Early (*using only pre-pregnancy features*)	Geographical	Test set	82,752 (0.15)	0.701 (0.696,0.706)	0.488 (0.478,0.498)
		Holdout set	5959 (0.20)	0.708 (0.69,0.725)	0.510 (0.475,0.544)
EHR-Early	Temporal	Test set	82,568 (0.12)	0.732 (0.727,0.737)	0.540 (0.529,0.550)
		Holdout set	5959 (0.20)	0.709 (0.692,0.727)	0.504 (0.471,0.537)
EHR-Early	Pooled 3-foldcross validation	Random test set	86,862 (0.13)	0.719 (0.716,0.722)	0.516 (0.511,0.522)
EHR-New onset*(pts without mental history)*	Pooled 3-foldcross validation	Random test set	67,105 (0.10)	0.666 (0.662–0.67)	0.413 (0.406–0.42)

**Fig. 4:**
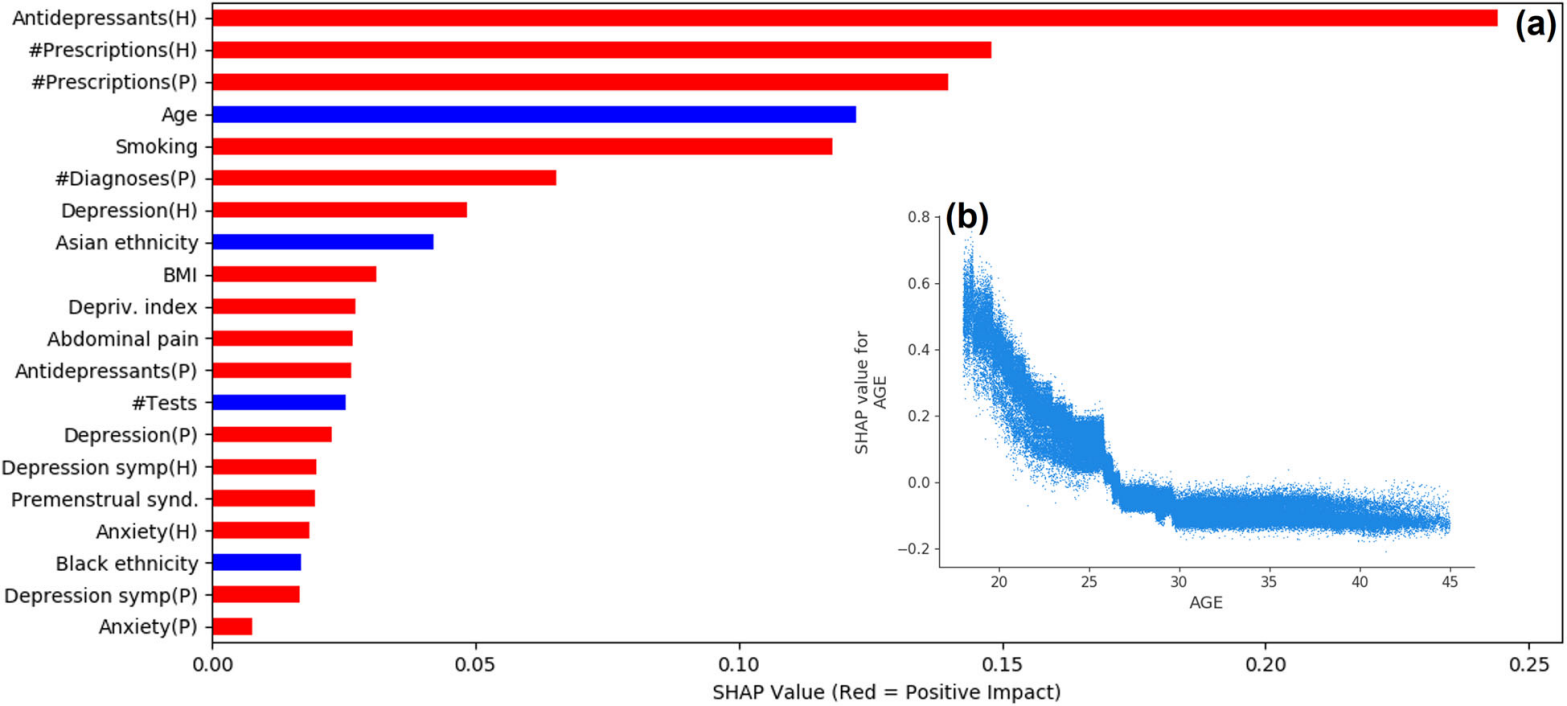
Feature significance using SHapley Additive exPlanations (SHAP). The top 20 contributing features from the time periods of the pregnancy (P) and the preceding two years (H). The red and blue bars indicate a positive and negative impact, respectively (**a**). For the age variable, the SHAP value per subject (**b**) indicates a stronger impact of age for younger patients

Analysis of predictor variable's contribution using SHAP (Fig. 4a) indicated that the total number of drug prescriptions before and during the pregnancy period had strong positive contribution to the predicted PPD risk score. Past prescriptions of antidepressants was the most prominent predictor. Among the medical diagnoses, the notable contributors were abdominal pain, premenstrual syndrome, previous depression and anxiety. Personal and demographic variables such as older age and non-white ethnicity contributed to a lower risk of PPD, while smoking, higher BMI and lower quantile of deprivation index increased the risk. A closer look on the per-patient contribution of the age variable (Fig. 4b) revealed that age was a significant variable in very young women (< 20 years old), and its significance decreased with age and reached a plateau in women older than 30 years old. This may imply that the risk of PPD is not linearly related to age, and the actual risk factor is giving birth at a very young age [24].

To evaluate the specific contribution of variables that are not related do mental health, we trained a prediction model without mental health variables (disease diagnoses, drug prescriptions and recorded symptoms of depression or anxiety). This model achieved AUC of 0.70, with the main contributing variables being utilization of healthcare services, including number of drug prescriptions, number of diagnoses and number of any lab tests. Other significant variables were age, smoking, deprivation index, pre-pregnancy BMI, ethnicity, history of premenstrual syndrome, abdominal pain and prescriptions of beta-blocking drugs. This type of model may be useful in clinical scenarios where the patients gets mental health services outside of their primary care health system.

## Discussion

PPD poses a serious threat on the wellbeing of the mother and the newborn child. From a public health perspective, it is a common complication of childbirth, with significant social implications and a large economic burden [25]. Although most of the risk factors for PPD are well-known, clinicians often overlook them due to lack of time and because patients may not fully disclose the symptoms of their mental illness. In addition, PPD risk is assessed by healthcare providers in a qualitative manner, which is limited by the human capabilities of integrating complex information. Machine learning models, on the other hand, can integrate a multitude of variables to provide a quantitative risk estimation, which may support the clinical decision making. Our results indicate that such integration of variables that are readily available in the patient’s primary care EHR is feasible and may enable fairly accurate risk estimation. EHR-based risk estimation can be executed automatically throughout the pregnancy and postnatal periods.

Primary care records provide rich representation of the continuous medical history and socio-demographic profile of the patients and are therefore an excellent data source for machine learning algorithms. Previous work on prediction of postpartum depression [16, 26] used hospital EHR data, which is typically limited to specific types of healthcare services (e.g. obstetrics), distinct periods of observation (e.g. pregnancy) and smaller cohorts of patients. Using retrospective analysis of primary care data, we were able to include a very large cohort of over 260K patients, and to identify PPD outcome more accurately from their follow-up medical records. Two additional new contributions of this work are the use of EHR data for early prediction of PPD, and the ability to use the predicted risk score to augment the diagnostic accuracy of routine PPD screening. As the holdout EPDS test set included women at higher risk of PPD, selected for evaluation by their physician, the predictive accuracy of EPDS score in this subgroup was probably higher than in a screening population, while the performance of the EHR-based prediction was similar to the larger test set (Fig. 3). It is therefore expected that the additive value of EHR-based prediction to EPDS will be higher when applied to a screening population. Additionally, our model achieved fair accuracy without using any mental health variables, implying that there are non-obvious associations between the predictor variables and the outcome, which may be utilized in cases where the full mental health history of the patient is not available. It should be noted that these associations do not imply casual effects between the predictor variables and the outcome. Our results are consistent with a recent study [17] that reported an AUC of 0.71 for an EHR-based PPD prediction model applied to an Israeli cohort of 214K women with PPD prevalence of 1.9%. Our cohort had significantly higher prevalence of PPD (13.4%) and included only first live births. Both models identified pre-gestational psychiatric disorders, smoking status and measures of healthcare utilization as major predictor variables. While this strengthens the validity of the models, it highlights the need for facilitating benchmarking and external validation of machine learning prediction models across multiple diverse data sources.

The incorporation of EHR-based prediction of PPD may facilitate early screening programs before the beginning of the pregnancy or during the first trimester. Women identified as having high risk of PPD may be offered closer follow-up during pregnancy, with possible treatment when appropriate. Later in pregnancy and following giving birth, EHR-scores may be combined with self-reported symptoms-based questionnaires such as EPDS to increase the sensitivity and the specificity of the screening process. This may also make the process more objective thus overcoming the inherent limitation of self-reported questionnaires.

Our study has several limitations – the PPD outcome definition was based on recorded diagnoses and treatments of depression from a primary care data source. The condition of depression may be under-recorded due to the stigmatic perception of mental health diseases, and because patients may be referred to depression counselling through perinatal mental health services, which may not be recorded by the primary care physician, resulting in possible misclassification of these patients. On the other hand, antidepressant drugs may be prescribed for indications other than depression (for example, anxiety or obsessive-compulsive disorders), so using them to derive the outcome may overestimate the occurrence of PPD. This is an inherent limitation of analyzing noisy, possible incomplete, real-world data. In addition, some of the relevant information may be missing from the EHR, or recorded in non-structured encounter notes, which were not available in this work. For example, ethnic group and marital status were only available in about half of the patient records. Ethnic minority and older age have been previously reported to increase the risk of PPD [27, 28]. Our result of lower-risk for non-white women may therefore indicate underdiagnosis in these population due to lack of awareness, fear of social stigma or communication problems [29]. Further study is required in order to build customized and fair prediction models for ethnically-heterogenous populations. Our cohort included only first livebirth pregnancies, thus overlooking the risk factor of PPD in a previous delivery, as well as the important goal of addressing depression following a still birth or an abortion. We intend to extend the analysis to these additional populations, and also to study the potential causal effect of timely treatment, initiated by early identification of PPD, on longer-term outcomes of the mother and child. Additional predictive variables, such as number of previous abortions, infant birth weight and infant feeding type may also contribute to the model’s performance. Another related research question is the transferability of prediction models between different health systems and populations, which is essential for further validation and for future deployment of such models in the clinical workflow.

## Conclusions

Data from electronic health records can be used for identifying women at risk of PPD. Our machine learning-based models achieved fair prediction performance and provided additive value to existing screening tools (EPDS). Furthermore, it allowed early alert of PPD risk prior to pregnancy. Incorporation of such models in the workflow of PPD screening may improve the subjectivity and accuracy of the screening process, enable timely interventions and consequently contribute to improved outcomes for the mother and child.

## Supplementary Information

**Additional file 1.** Supplementary Appendix.

## Data Availability

The raw data that support the findings of this study are available from IQVIA Inc. but restrictions apply to the availability of these data, which were used under license for the current study, and so are not publicly available. Data are however available from the authors upon reasonable request and with permission of IQVIA Inc.
