# Supplementary material for: Estimation of postpartum depression risk from electronic health records using machine learning
Source: BMC Pregnancy Childbirth. 2021 Sep 17;21:630. doi: 10.1186/s12884-021-04087-8 (PMC8447665; doi:10.1186/s12884-021-04087-8)
Supplement: Supplementary file 1 — Additional file 1. Supplementary Appendix. [file 12884_2021_4087_MOESM1_ESM.docx]

**Estimation of Postpartum Depression Risk from
Electronic Health Records Using Machine Learning**

Guy Amit^1†^, Irena Girshovitz^1^, Karni Marcus^1^, Yiye Zhang^2^, Jyotishman Pathak^2^,
Vered Bar^3^, Pinchas Akiva^1^

^1^ KI Research Institute, Kfar Malal, Israel
^2^ Weill Cornell Medicine, Healthcare Policy and Research, New York, NY

^3^ Sheba Medical Center, Women Mental Health, Ramat Gan, Israel

^†^ Corresponding author

**Supplementary Appendix**

1. Read codes of outcome and predictor variables:

**Depression:** E112200, Eu92000, E112500, E113z00, E2B..00, E112.11, E204.11, Eu32.13, Eu33.14, E113700, E113300, Eu33.12, Eu33.00, Eu32B00, Eu32z13, Eu32.00, 8BK0.00, Eu32z14, E112000, Eu32213, Eu32.11, Eu32y00, Eu33z11, Eu33211, Eu32211, Eu41200, Eu34113, Eu34114, E112.14, Eu34.00, E11..12, Eu33.15, Eu32z12, Eu32900, E113000, Eu33214, Eu32A00, Eu32700, Eu32z11, Eu32500, E291.00, Eu32212, Eu32y11, Eu41211, E113100, Eu33100, E2B1.00, E11z200, Eu34y00, Eu32400, Eu32000, 62T1.00, Eu32600, E113.11, Eu32y12, Eu53011, Eu33y00, Eu33000, Eu33200, 8CAa.00, E211200, E11y200, 8HHq.00, Eu32314, Eu33.13, E204.00, E112.13, E113200, E113.00, E135.00, Eu34111, Eu33.11, E113500, E112100, Eu33212, E112.00, Eu32.12, Eu32100, Eu3y111, E200300, Eu53012, Eu34z00, E112.12, 6G00.00, R007z13, Eu32200, E118.00, Eu32z00, Eu33z00, E112300, E2B0.00, E112z00, E130.11, Eu33313, E113400, Eu32312, Eu33300, Eu33316, Eu32311, Eu32300, E112400, Eu33311, Eu33315, E130.00, Eu32800, Eu32313, Eu33314

**Non-pharmacological treatment for depression:** Z523.11, Z523.12, Z523100, Z523200, Z523211, Z523300, 9N1T.00, 8H49.00, ZL9D.00, 665..00, 8CM2.00, 9N1M.00, 6779.00, 8H7T.00, 6654.00, 9N2W.00, ZL5B.00, 8A2..00, 8H23.00, ZLBB.00, 9N2a.00, 9NN5.00, ZL78.11, 9NNM.00, 9Nla.00, ZLBC.00, ZL78.00, ZL1B.00, 8G51.00, 8HVO.00, 8HHT.00, 9NlK.00, ZL77.00, Z4L..00

**Anxiety:** E202A00, Eu40213, E202C00, E202500, E202000, E202200, Eu40z12, E202.00, Eu40212, E202900, E202600, Eu40011, Eu40100, E202.11, E202z11, E202700, E202300

**Anxiety symptoms:** 1B13.11, 1B12.12, 1B12.11, 1B13.00, 1BK..00, 1B12.00, 2258.00, 1B13.12

**Depression symptoms:** 1B17.00, 1BP0.00, 1BT..00, 1BT..11, 2257.00, 1BI..00, 1BU..00, 1B17.11, 1BT..12, 1B1U.11, 1BQ..00, 1B1U.00

**Other mental disorders:** Eu20.00, E1...00, E110.11, Eu30100, E116.00, Eu31000, 1S42.00, E110000, 1BH..11, Eu33311, Eu30.11, Eu22012, E113400, Eu30200, Eu22000, E130.00, R001z00, Eu33300, Eu20000, E100000, E23..00, E24..00, E25..00, E29..00, E21..00, E21..00, E21y200

**Premenstrual syndrome:** K584.00, Eu3y200

Preeclampsia: L124600, L125.00, L124.11, L129.00, L124.12, L126.00, L124.00, L124z00, L12B.00, L124100, L124000, L126500, L126300, L125100, L125z00, L127100, L124300, L126600, L127000, L126000, L126100, L125000, L127.00, L125300, L126400, L126z00, L125400

**Gestational diabetes**: L180811, L180.00, L180800, L180z00, L180300, L180100

**Hypertension**: G22..00, G24zz00, 662b.00, G200.00, 9OIA.11, 9h3..00, L128200, 9OI7.00, TJC7.00, G241z00, Gyu2000, G202.00, G21z011, 662P.00, G20..00, G2…00, G20..11, 9OI4.00, 9OIA.00, G20z.00, G20z.11, 662d.00, 9N1y200, G2z..00, 246M.00, 9OI5.00, 9OI..00, 662G.00

**Pregnancy vomiting**: L13.x

**Migraine**: 1474.00, 1474000, 1967.00, 8B6N.00, F26..00, F260.00, F260.11, F261.00, F261000, F261.11, F261z00, F262.00, F262200, F262300, F262400, F262500, F262800, F262z00, F26y.00, F26y000, F26y100, F26y111, F26y200, F26y300, F26yz00, F26z.00, Fyu5300, K584.11, R090D00

**Diarrhea**: 19F..00, 19F..11, 19F2.00, 19FZ.00, 19FZ.11, 19G..00, J525.00, J43z.11, J4zz.11, A083.11, A083.00, A076.11, A082.00, A082z00, J4...13

**Abdominal pain**: 197C.00, 1971.00, R090N00, 197A.00, 197B.00, R090E00, R090H00, 197A.11, R090y00, R090P00, Ryu1100, 1969.00, R090.00, R090z00, L16y500

**C-section**: 14Y0.00, 14Y2.00, 14Y6.00, 7F12.00, 7F12000, 7F12100, 7F12111, 7F12y00, 7F12z00, 7F13.00, 7F13000, 7F13100, 7F13111, 7F13200, 7F13300, 7F13y00, 7F13z00, 7F1A000, L213200, L398.00, L398000, L398100, L398200, L398300, L398400, L398500, L398600, L398z00, L441.00, L441000, L441100, L441200, L441z00, Lyu5200, Lyu6A00, Q021300, Q034.00, Z254500, Z254600

**Obesity**: 222A.00, 22A5.11, 22K5.00, 22K7.00, 66C..00, 66C1.00, 66C2.00, 66C4.00, 66C5.00, 66C6.00, 66C7.00, 66CE.00, 66CZ.00, 9OK..00, 9OK..11, 9OK1.00, 9OK2.00, 9OK3.00, 9OK4.00, 9OK5.00, 9OK6.00, 9OK7.00, 9OK8.00, 9OK9.00, 9OKA.00, 9OKZ.00, 9hN..00, 9hN0.00, 9hN1.00, C380.00, C380000, C380100, C380200, C380300, C380400, C380500, C380600, C380700, C38y011, C38z000, Cyu7000, ZC2CM00, ZV65319

**Drug abuse**: 8B23.00, 46QB.00, 46QA.00, E24..00, 46Q5.00, E24z.00, 8B23.12, 8B23.11, E240.00, ZV6D700, 46QH.00, 46Qf.00, 46Q5.11, E240.11, E24..11, 13c..00, E25z.00, Eu11212, 1463.00, E020.00, 8HHe.00, 9NN1.00, 146F.00, 8BA9.00, E240z00, 1J1..00, 8H7x.00, 9N0Z.00, E25..00, Eu11211, 46Qr.00, E252.00, 44uK.00, E243.00, ZV4K100, 8B2P.00, E240.12, E244.00, 13cM.00, E242.00, Eu12211, 1T8..00, 9OhB.00, L183.00, ZG23200, Eu19211, 146E.00, ZV57B00

**Alcohol abuse**: 1365.00, 1366.00, 1369.00, 136P.00, 136Q.00, 136S.00, 136 T.0, 136 W.0, 136Y.00, 13Y8.00, 66e..00, 7P22100, 8BA8.00, 8H7p.00, 8HHe.00, 9NN2.00, 9k1..00, E01y000, E23..00, E23..11, E23..12, E230.00, E231.00, E231000, E231100, E231200, E231300, E231z00, E23z.00, E250.00, E250.14, E250000, E250200, Eu10011, Eu10211, J153.00, R103.00,

1. Codes of drug prescriptions:

**Antidepressants**: ATC N06A, excluding the following double-indication drugs [26]:

Amitriptyline/Tryptizol drug codes 70290979, 81024979, 99472998, 99824992, 94703998, 94077990, 96328979, 97223998, 99861990, 99863990, 99864990, 99866990, 99868990, 99869990, 99870990, 99871990, 81085998, 47944978, 81084998, 92808996, 98067988, 98128998, 96924998, 94704998, 99826992, 94704997, 98343998, 94703997, 95574997, 99017998, 96925998, 94076990, 94771990, 96323979, 97223997, 99861989, 99862990, 99863989, 99864989, 99865990, 99866989, 99867989, 99868989, 99869988, 99870989, 99871989, 92808997, 96891992, 98067990, 99825992, 96925997, 94075990, 97223996, 99863988, 99864988, 99866988, 99868988, 99869989, 99870988, 99871988, 92808998, 98067989, 94067992, 96925996, 83620998, 99017997, 98129998, 98130998, 98150998, 98138998, 98130997, 98150997, 98138997, 98130996, 98150996, 98129997

Anafranil/Clomipramine drug codes 80548979, 96640998, 97548990, 98144998, 98340990, 99297990, 97167992, 96640997, 96901989, 97548989, 97773989, 98144997, 98340989, 99297989, 96639998, 98143998, 80550979, 93358992, 96640996, 96901988, 97548988, 98144996, 98340988, 99297988, 83878998, 96638998, 93360992, 96637998, 99794992, 98142998

Cymbalta/Duloxetine/Yentreve drug codes 39667978, 86997998, 86999998, 89023979, 37600978, 51109978, 86996998, 86998998

Fluanxol/Flupentixol drug codes 96504997, 99634997, 96504998, 99634998

Nortriptyline drug codes 64091979, 94249992, 94630998, 95695998, 92015990, 95696998, 96248979, 98152998, 95695996, 95695997, 92014990, 95696997, 96244979, 98152997, 94630997, 98154998, 98154996, 98154997

**Antihistamines**: ATC R06A

**Antibacterials**: ATC J01X

**Beta blockers**: ATC C07A

1. Variables included in the prediction models

| No. | Variable Name |
| --- | --- |
| General | |
|  | Age |
|  | Pre-pregnancy BMI |
|  | Marital status - single |
|  | Marital status - married |
|  | Marital status - unknown |
|  | Deprivation index |
|  | Season in year |
|  | Smoking |
|  | Ethnicity - white |
|  | Ethnicity - Asian |
|  | Ethnicity - black |
|  | Ethnicity - mixed |
|  | Ethnicity - other |
|  | Ethnicity - unknown |
| Diagnoses during pregnancy | |
|  | Anxiety |
|  | Anxiety symptoms |
|  | Depression |
|  | Depression symptoms |
|  | Other mental disorders |
|  | Pregnancy complication |
|  | Candidal vulvovaginitis |
|  | Vomiting |
|  | Threatened miscarriage |
|  | Palpitations |
|  | Obesity |
|  | Gestational diabetes mellitus |
|  | Preeclampsia |
|  | Hypothyroidism |
|  | Hypertension |
|  | pharyngitis |
|  | Migraine |
|  | Diarrhea |
|  | Abdominal pain |
|  | Placental infarct |
|  | Hemorrhage |
|  | Abnormality of organs |
|  | False labor |
| Drug prescriptions during pregnancy | |
|  | Antidepressants |
|  | Thyroid preparations |
|  | Antihistamines for systemic use |
|  | Beta blocking agents |
|  | Direct acting antivirals |
|  | Other antibacterials |
| Other (pregnancy and labor) | |
|  | Primigravida |
|  | Cesarean section |
|  | Gestational week |
|  | Low platelets |
|  | Weight (2^nd^ trimester) |
|  | Diastolic blood pressure (3^rd^ trimester) |
|  | Number of ED visits |
|  | Number of diagnoses |
|  | Number of drug prescriptions |
|  | Number of lab tests |
| Medical history before pregnancy | |
|  | Anxiety |
|  | Anxiety symptoms |
|  | Depression |
|  | Depression symptoms |
|  | Other mental disorders |
|  | Premenstrual syndrome |
|  | Alcohol abuse |
|  | Drug abuse |
|  | Prescription of Antidepressants |
|  | Prescription of Thyroid preparations |
|  | Prescription of Antihistamines |
|  | Prescription of Beta blocking agents |
|  | Prescription of antibacterial |
|  | Number of diagnoses |
|  | Number of drug prescriptions |
|  | Number of lab tests |
